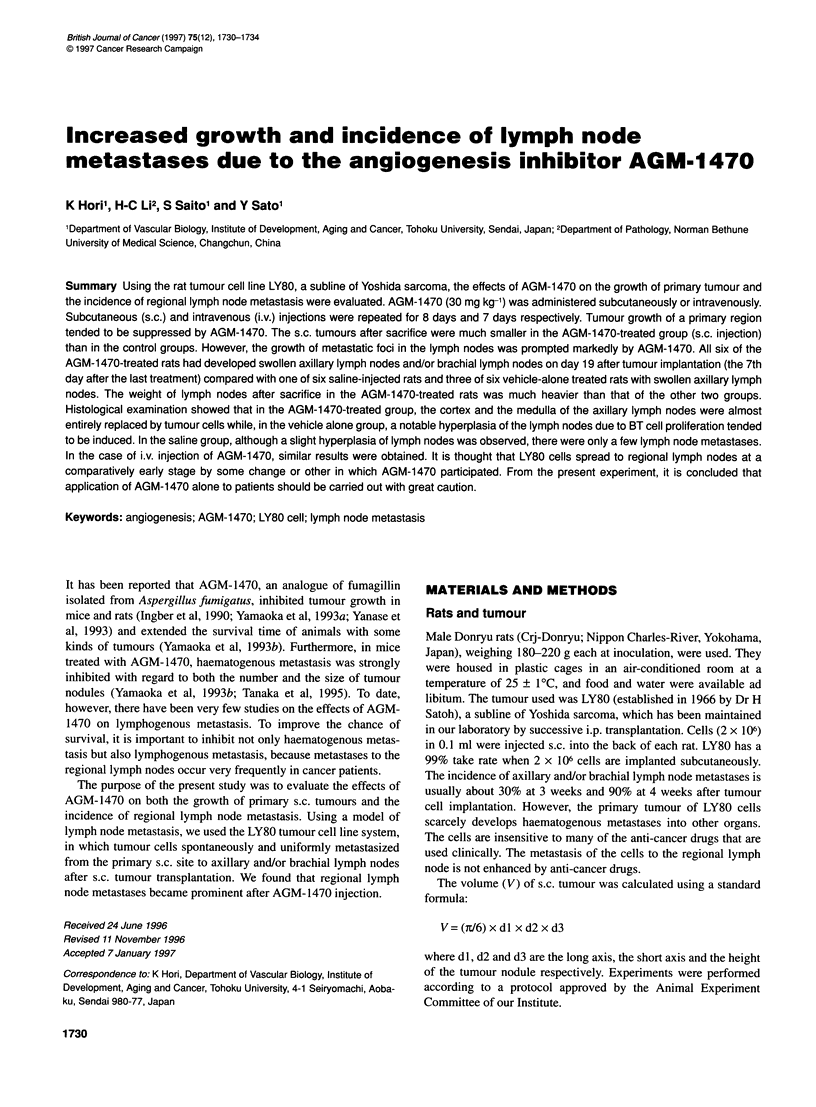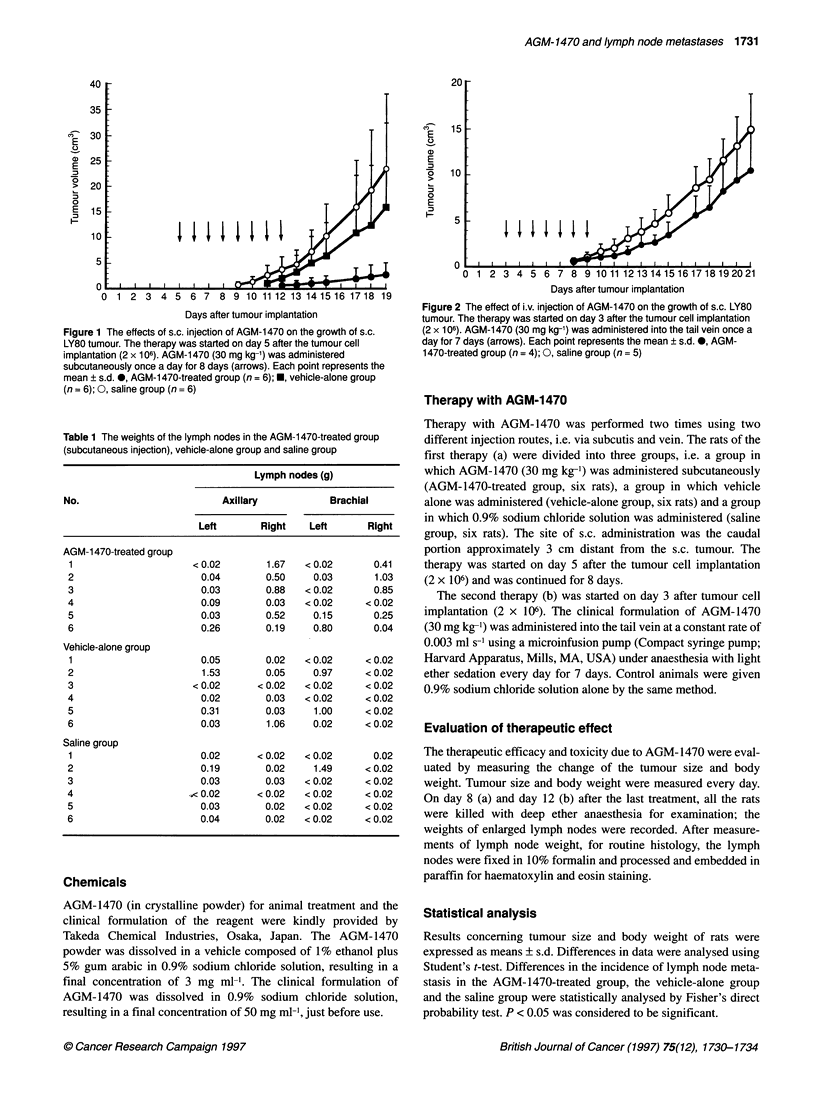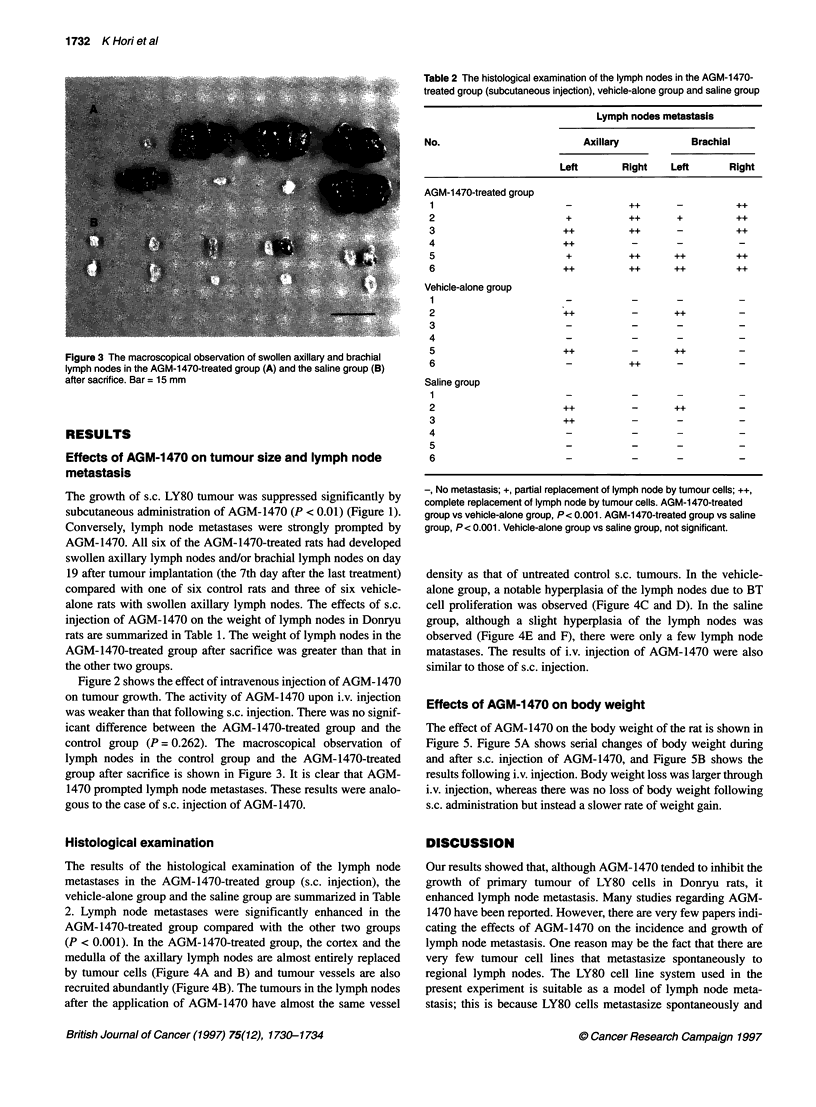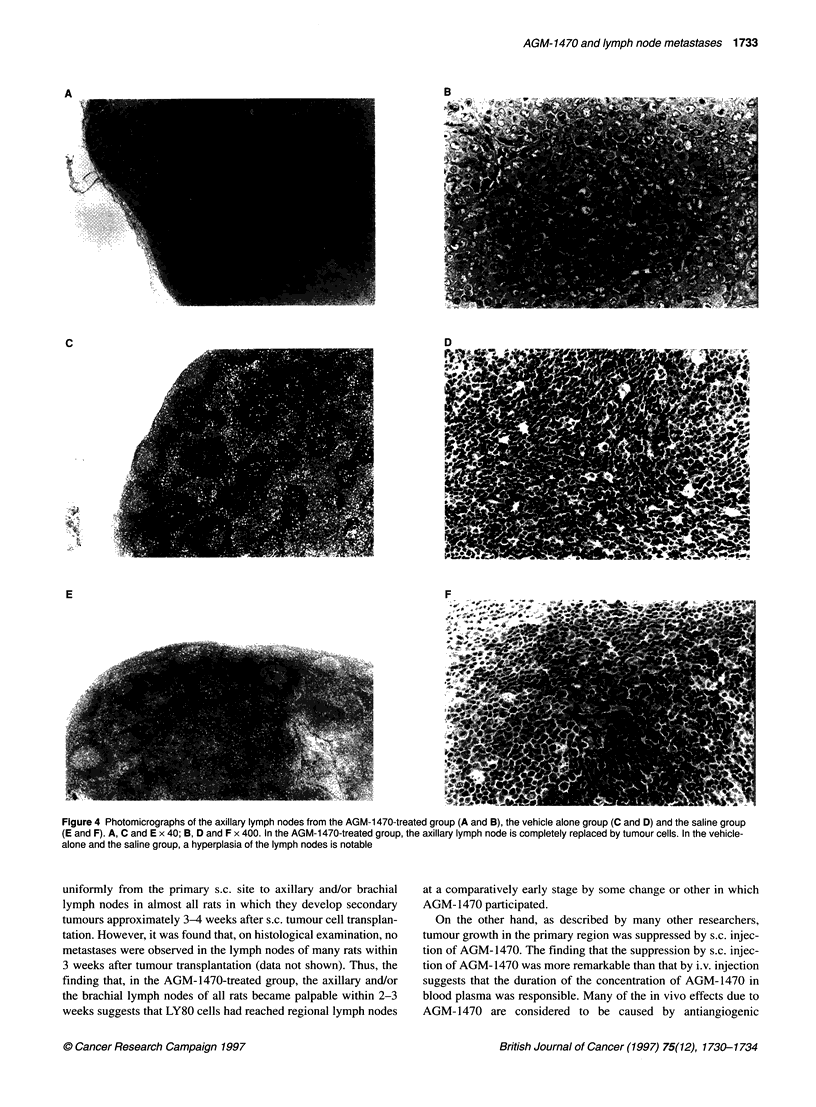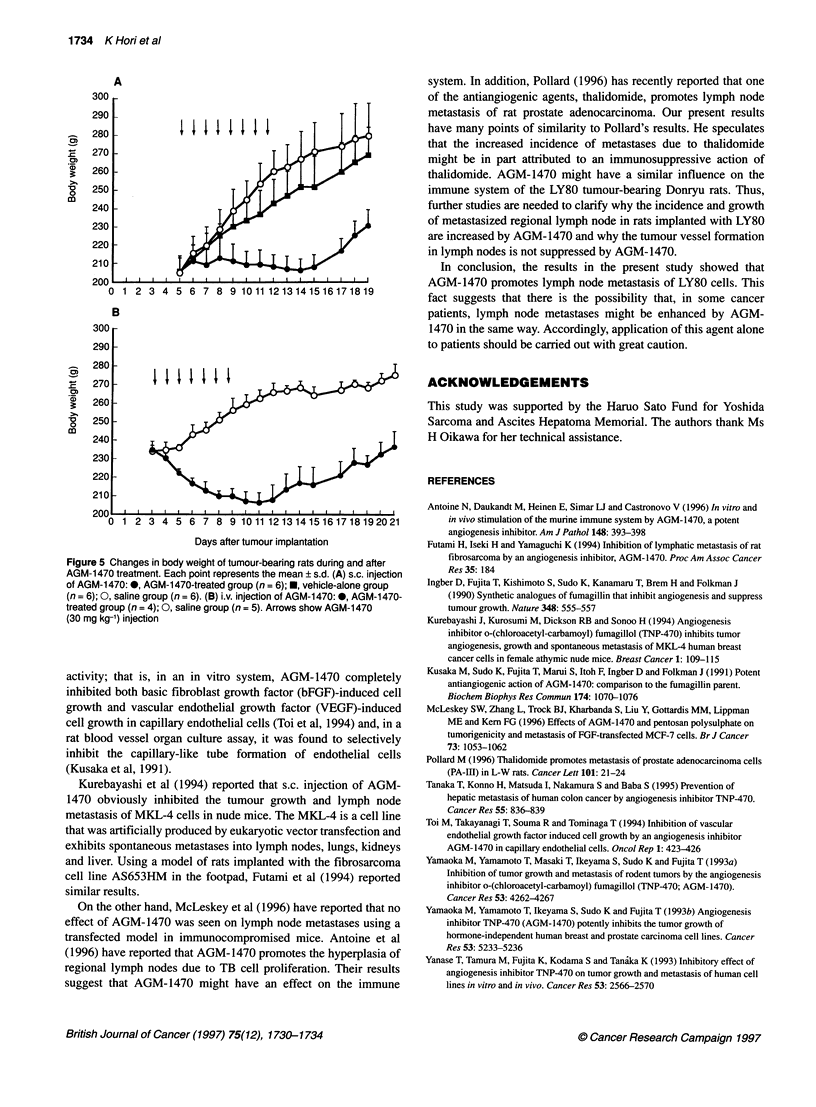# Increased growth and incidence of lymph node metastases due to the angiogenesis inhibitor AGM-1470.

**DOI:** 10.1038/bjc.1997.296

**Published:** 1997

**Authors:** K. Hori, H. C. Li, S. Saito, Y. Sato

**Affiliations:** Department of Vascular Biology, Institute of Development, Aging and Cancer, Tohoku University, Sendai, Japan.

## Abstract

**Images:**


British Journal of Cancer (1997) 75(12), 1730–1734
© 1997 Cancer Research Campaign

### Increased growth and incidence of lymph node metastases due to the angiogenesis inhibitor AGM-1470

**K Hori[1], H-C Li[2], S Saito[1] and Y Sato[1]**

[1]Department of Vascular Biology, Institute of Development, Aging and Cancer, Tohoku University, Sendai, Japan; [2]Department of Pathology, Norman Bethune University of Medical Science, Changchun, China

**Summary** Using the rat tumour cell line LY80, a subline of Yoshida sarcoma, the effects of AGM-1470 on the growth of primary tumour and the incidence of regional lymph node metastasis were evaluated. AGM-1470 (30 mg $kg^{-1}$) was administered subcutaneously or intravenously. Subcutaneous (s.c.) and intravenous (i.v.) injections were repeated for 8 days and 7 days respectively. Tumour growth of a primary region tended to be suppressed by AGM-1470. The s.c. tumours after sacrifice were much smaller in the AGM-1470-treated group (s.c. injection) than in the control groups. However, the growth of metastatic foci in the lymph nodes was prompted markedly by AGM-1470. All six of the AGM-1470-treated rats had developed swollen axillary lymph nodes and/or brachial lymph nodes on day 19 after tumour implantation (the 7th day after the last treatment) compared with one of six saline-injected rats and three of six vehicle-alone treated rats with swollen axillary lymph nodes. The weight of lymph nodes after sacrifice in the AGM-1470-treated rats was much heavier than that of the other two groups. Histological examination showed that in the AGM-1470-treated group, the cortex and the medulla of the axillary lymph nodes were almost entirely replaced by tumour cells while, in the vehicle alone group, a notable hyperplasia of the lymph nodes due to BT cell proliferation tended to be induced. In the saline group, although a slight hyperplasia of lymph nodes was observed, there were only a few lymph node metastases. In the case of i.v. injection of AGM-1470, similar results were obtained. It is thought that LY80 cells spread to regional lymph nodes at a comparatively early stage by some change or other in which AGM-1470 participated. From the present experiment, it is concluded that application of AGM-1470 alone to patients should be carried out with great caution.

**Keywords:** angiogenesis; AGM-1470; LY80 cell; lymph node metastasis

It has been reported that AGM-1470, an analogue of fumagillin isolated from *Aspergillus fumigatus*, inhibited tumour growth in mice and rats (Ingber et al, 1990; Yamaoka et al, 1993*a*; Yanase et al, 1993) and extended the survival time of animals with some kinds of tumours (Yamaoka et al, 1993*b*). Furthermore, in mice treated with AGM-1470, haematogenous metastasis was strongly inhibited with regard to both the number and the size of tumour nodules (Yamaoka et al, 1993*b*; Tanaka et al, 1995). To date, however, there have been very few studies on the effects of AGM-1470 on lymphogenous metastasis. To improve the chance of survival, it is important to inhibit not only haematogenous metastasis but also lymphogenous metastasis, because metastases to the regional lymph nodes occur very frequently in cancer patients.

The purpose of the present study was to evaluate the effects of AGM-1470 on both the growth of primary s.c. tumours and the incidence of regional lymph node metastasis. Using a model of lymph node metastasis, we used the LY80 tumour cell line system, in which tumour cells spontaneously and uniformly metastasized from the primary s.c. site to axillary and/or brachial lymph nodes after s.c. tumour transplantation. We found that regional lymph node metastases became prominent after AGM-1470 injection.

*Received 24 June 1996*
*Revised 11 November 1996*
*Accepted 7 January 1997*

*Correspondence to:* K Hori, Department of Vascular Biology, Institute of Development, Aging and Cancer, Tohoku University, 4-1 Seiryomachi, Aoba-ku, Sendai 980-77, Japan

### MATERIALS AND METHODS

### Rats and tumour

Male Donryu rats (Crj-Donryu; Nippon Charles-River, Yokohama, Japan), weighing 180–220 g each at inoculation, were used. They were housed in plastic cages in an air-conditioned room at a temperature of 25 ± 1°C, and food and water were available ad libitum. The tumour used was LY80 (established in 1966 by Dr H Satoh), a subline of Yoshida sarcoma, which has been maintained in our laboratory by successive i.p. transplantation. Cells ($2 \times 10^6$) in 0.1 ml were injected s.c. into the back of each rat. LY80 has a 99% take rate when $2 \times 10^6$ cells are implanted subcutaneously. The incidence of axillary and/or brachial lymph node metastases is usually about 30% at 3 weeks and 90% at 4 weeks after tumour cell implantation. However, the primary tumour of LY80 cells scarcely develops haematogenous metastases into other organs. The cells are insensitive to many of the anti-cancer drugs that are used clinically. The metastasis of the cells to the regional lymph node is not enhanced by anti-cancer drugs.

The volume ($V$) of s.c. tumour was calculated using a standard formula:

$$V = (\pi/6) \times d1 \times d2 \times d3$$

where d1, d2 and d3 are the long axis, the short axis and the height of the tumour nodule respectively. Experiments were performed according to a protocol approved by the Animal Experiment Committee of our Institute.

**Figure 1** The effects of s.c. injection of AGM-1470 on the growth of s.c. LY80 tumour. The therapy was started on day 5 after the tumour cell implantation ($2 \times 10^6$). AGM-1470 (30 mg $kg^{-1}$) was administered subcutaneously once a day for 8 days (arrows). Each point represents the mean ± s.d. ●, AGM-1470-treated group (*n* = 6); ■, vehicle-alone group (*n* = 6); ○, saline group (*n* = 6)

**Table 1** The weights of the lymph nodes in the AGM-1470-treated group (subcutaneous injection), vehicle-alone group and saline group

| No. | Lymph nodes (g) | | | |
|---|---|---|---|---|
| | Axillary | | Brachial | |
| | Left | Right | Left | Right |
| AGM-1470-treated group | | | | |
| 1 | < 0.02 | 1.67 | < 0.02 | 0.41 |
| 2 | 0.04 | 0.50 | 0.03 | 1.03 |
| 3 | 0.03 | 0.88 | < 0.02 | 0.85 |
| 4 | 0.09 | 0.03 | < 0.02 | < 0.02 |
| 5 | 0.03 | 0.52 | 0.15 | 0.25 |
| 6 | 0.26 | 0.19 | 0.80 | 0.04 |
| Vehicle-alone group | | | | |
| 1 | 0.05 | 0.02 | < 0.02 | < 0.02 |
| 2 | 1.53 | 0.05 | 0.97 | < 0.02 |
| 3 | < 0.02 | < 0.02 | < 0.02 | < 0.02 |
| 4 | 0.02 | 0.03 | < 0.02 | < 0.02 |
| 5 | 0.31 | 0.03 | 1.00 | < 0.02 |
| 6 | 0.03 | 1.06 | 0.02 | < 0.02 |
| Saline group | | | | |
| 1 | 0.02 | < 0.02 | < 0.02 | 0.02 |
| 2 | 0.19 | 0.02 | 1.49 | < 0.02 |
| 3 | 0.03 | 0.03 | < 0.02 | < 0.02 |
| 4 | < 0.02 | < 0.02 | < 0.02 | < 0.02 |
| 5 | 0.03 | 0.02 | < 0.02 | < 0.02 |
| 6 | 0.04 | 0.02 | < 0.02 | < 0.02 |

### Chemicals

AGM-1470 (in crystalline powder) for animal treatment and the clinical formulation of the reagent were kindly provided by Takeda Chemical Industries, Osaka, Japan. The AGM-1470 powder was dissolved in a vehicle composed of 1% ethanol plus 5% gum arabic in 0.9% sodium chloride solution, resulting in a final concentration of 3 mg $ml^{-1}$. The clinical formulation of AGM-1470 was dissolved in 0.9% sodium chloride solution, resulting in a final concentration of 50 mg $ml^{-1}$, just before use.

**Figure 2** The effect of i.v. injection of AGM-1470 on the growth of s.c. LY80 tumour. The therapy was started on day 3 after the tumour cell implantation ($2 \times 10^6$). AGM-1470 (30 mg $kg^{-1}$) was administered into the tail vein once a day for 7 days (arrows). Each point represents the mean ± s.d. ●, AGM-1470-treated group (*n* = 4); ○, saline group (*n* = 5)

### Therapy with AGM-1470

Therapy with AGM-1470 was performed two times using two different injection routes, i.e. via subcutis and vein. The rats of the first therapy (a) were divided into three groups, i.e. a group in which AGM-1470 (30 mg $kg^{-1}$) was administered subcutaneously (AGM-1470-treated group, six rats), a group in which vehicle alone was administered (vehicle-alone group, six rats) and a group in which 0.9% sodium chloride solution was administered (saline group, six rats). The site of s.c. administration was the caudal portion approximately 3 cm distant from the s.c. tumour. The therapy was started on day 5 after the tumour cell implantation ($2 \times 10^6$) and was continued for 8 days.

The second therapy (b) was started on day 3 after tumour cell implantation ($2 \times 10^6$). The clinical formulation of AGM-1470 (30 mg $kg^{-1}$) was administered into the tail vein at a constant rate of 0.003 ml $s^{-1}$ using a microinfusion pump (Compact syringe pump; Harvard Apparatus, Mills, MA, USA) under anaesthesia with light ether sedation every day for 7 days. Control animals were given 0.9% sodium chloride solution alone by the same method.

### Evaluation of therapeutic effect

The therapeutic efficacy and toxicity due to AGM-1470 were evaluated by measuring the change of the tumour size and body weight. Tumour size and body weight were measured every day. On day 8 (a) and day 12 (b) after the last treatment, all the rats were killed with deep ether anaesthesia for examination; the weights of enlarged lymph nodes were recorded. After measurements of lymph node weight, for routine histology, the lymph nodes were fixed in 10% formalin and processed and embedded in paraffin for haematoxylin and eosin staining.

### Statistical analysis

Results concerning tumour size and body weight of rats were expressed as means ± s.d. Differences in data were analysed using Student's *t*-test. Differences in the incidence of lymph node metastasis in the AGM-1470-treated group, the vehicle-alone group and the saline group were statistically analysed by Fisher's direct probability test. $P < 0.05$ was considered to be significant.

© Cancer Research Campaign 1997

**Figure 3** The macroscopical observation of swollen axillary and brachial lymph nodes in the AGM-1470-treated group (**A**) and the saline group (**B**) after sacrifice. Bar = 15 mm

### RESULTS

### Effects of AGM-1470 on tumour size and lymph node metastasis

The growth of s.c. LY80 tumour was suppressed significantly by subcutaneous administration of AGM-1470 ($P < 0.01$) (Figure 1). Conversely, lymph node metastases were strongly prompted by AGM-1470. All six of the AGM-1470-treated rats had developed swollen axillary lymph nodes and/or brachial lymph nodes on day 19 after tumour implantation (the 7th day after the last treatment) compared with one of six control rats and three of six vehicle-alone rats with swollen axillary lymph nodes. The effects of s.c. injection of AGM-1470 on the weight of lymph nodes in Donryu rats are summarized in Table 1. The weight of lymph nodes in the AGM-1470-treated group after sacrifice was greater than that in the other two groups.

Figure 2 shows the effect of intravenous injection of AGM-1470 on tumour growth. The activity of AGM-1470 upon i.v. injection was weaker than that following s.c. injection. There was no significant difference between the AGM-1470-treated group and the control group ($P = 0.262$). The macroscopical observation of lymph nodes in the control group and the AGM-1470-treated group after sacrifice is shown in Figure 3. It is clear that AGM-1470 prompted lymph node metastases. These results were analogous to the case of s.c. injection of AGM-1470.

### Histological examination

The results of the histological examination of the lymph node metastases in the AGM-1470-treated group (s.c. injection), the vehicle-alone group and the saline group are summarized in Table 2. Lymph node metastases were significantly enhanced in the AGM-1470-treated group compared with the other two groups ($P < 0.001$). In the AGM-1470-treated group, the cortex and the medulla of the axillary lymph nodes are almost entirely replaced by tumour cells (Figure 4A and B) and tumour vessels are also recruited abundantly (Figure 4B). The tumours in the lymph nodes after the application of AGM-1470 have almost the same vessel density as that of untreated control s.c. tumours. In the vehicle-alone group, a notable hyperplasia of the lymph nodes due to BT cell proliferation was observed (Figure 4C and D). In the saline group, although a slight hyperplasia of the lymph nodes was observed (Figure 4E and F), there were only a few lymph node matastases. The results of i.v. injection of AGM-1470 were also similar to those of s.c. injection.

**Table 2** The histological examination of the lymph nodes in the AGM-1470-treated group (subcutaneous injection), vehicle-alone group and saline group

| No. | Lymph nodes metastasis | | | |
|---|---|---|---|---|
| | Axillary | | Brachial | |
| | Left | Right | Left | Right |
| AGM-1470-treated group | | | | |
| 1 | – | ++ | – | ++ |
| 2 | + | ++ | + | ++ |
| 3 | ++ | ++ | – | ++ |
| 4 | ++ | – | – | – |
| 5 | + | ++ | ++ | ++ |
| 6 | ++ | ++ | ++ | ++ |
| Vehicle-alone group | | | | |
| 1 | – | – | – | – |
| 2 | ++ | – | ++ | – |
| 3 | – | – | – | – |
| 4 | – | – | – | – |
| 5 | ++ | – | ++ | – |
| 6 | – | ++ | – | – |
| Saline group | | | | |
| 1 | – | – | – | – |
| 2 | ++ | – | ++ | – |
| 3 | ++ | – | – | – |
| 4 | – | – | – | – |
| 5 | – | – | – | – |
| 6 | – | – | – | – |

–, No metastasis; +, partial replacement of lymph node by tumour cells; ++, complete replacement of lymph node by tumour cells. AGM-1470-treated group vs vehicle-alone group, $P < 0.001$. AGM-1470-treated group vs saline group, $P < 0.001$. Vehicle-alone group vs saline group, not significant.

### Effects of AGM-1470 on body weight

The effect of AGM-1470 on the body weight of the rat is shown in Figure 5. Figure 5A shows serial changes of body weight during and after s.c. injection of AGM-1470, and Figure 5B shows the results following i.v. injection. Body weight loss was larger through i.v. injection, whereas there was no loss of body weight following s.c. administration but instead a slower rate of weight gain.

### DISCUSSION

Our results showed that, although AGM-1470 tended to inhibit the growth of primary tumour of LY80 cells in Donryu rats, it enhanced lymph node metastasis. Many studies regarding AGM-1470 have been reported. However, there are very few papers indicating the effects of AGM-1470 on the incidence and growth of lymph node metastasis. One reason may be the fact that there are very few tumour cell lines that metastasize spontaneously to regional lymph nodes. The LY80 cell line system used in the present experiment is suitable as a model of lymph node metastasis; this is because LY80 cells metastasize spontaneously and

*© Cancer Research Campaign 1997*

**Figure 4** Photomicrographs of the axillary lymph nodes from the AGM-1470-treated group (**A** and **B**), the vehicle alone group (**C** and **D**) and the saline group (**E** and **F**). **A**, **C** and **E** × 40; **B**, **D** and **F** × 400. In the AGM-1470-treated group, the axillary lymph node is completely replaced by tumour cells. In the vehicle-alone and the saline group, a hyperplasia of the lymph nodes is notable uniformly from the primary s.c. site to axillary and/or brachial lymph nodes in almost all rats in which they develop secondary tumours approximately 3–4 weeks after s.c. tumour cell transplantation. However, it was found that, on histological examination, no metastases were observed in the lymph nodes of many rats within 3 weeks after tumour transplantation (data not shown). Thus, the finding that, in the AGM-1470-treated group, the axillary and/or the brachial lymph nodes of all rats became palpable within 2–3 weeks suggests that LY80 cells had reached regional lymph nodes at a comparatively early stage by some change or other in which AGM-1470 participated.

On the other hand, as described by many other researchers, tumour growth in the primary region was suppressed by s.c. injection of AGM-1470. The finding that the suppression by s.c. injection of AGM-1470 was more remarkable than that by i.v. injection suggests that the duration of the concentration of AGM-1470 in blood plasma was responsible. Many of the in vivo effects due to AGM-1470 are considered to be caused by antiangiogenic

© Cancer Research Campaign 1997

**Figure 5** Changes in body weight of tumour-bearing rats during and after AGM-1470 treatment. Each point represents the mean ± s.d. (**A**) s.c. injection of AGM-1470: ●, AGM-1470-treated group (*n* = 6); ■, vehicle-alone group (*n* = 6); ○, saline group (*n* = 6). (**B**) i.v. injection of AGM-1470: ●, AGM-1470-treated group (*n* = 4); ○, saline group (*n* = 5). Arrows show AGM-1470 (30 mg $kg^{-1}$) injection activity; that is, in an in vitro system, AGM-1470 completely inhibited both basic fibroblast growth factor (bFGF)-induced cell growth and vascular endothelial growth factor (VEGF)-induced cell growth in capillary endothelial cells (Toi et al, 1994) and, in a rat blood vessel organ culture assay, it was found to selectively inhibit the capillary-like tube formation of endothelial cells (Kusaka et al, 1991).

Kurebayashi et al (1994) reported that s.c. injection of AGM-1470 obviously inhibited the tumour growth and lymph node metastasis of MKL-4 cells in nude mice. The MKL-4 is a cell line that was artificially produced by eukaryotic vector transfection and exhibits spontaneous metastases into lymph nodes, lungs, kidneys and liver. Using a model of rats implanted with the fibrosarcoma cell line AS653HM in the footpad, Futami et al (1994) reported similar results.

On the other hand, McLeskey et al (1996) have reported that no effect of AGM-1470 was seen on lymph node metastases using a transfected model in immunocompromised mice. Antoine et al (1996) have reported that AGM-1470 promotes the hyperplasia of regional lymph nodes due to TB cell proliferation. Their results suggest that AGM-1470 might have an effect on the immune system. In addition, Pollard (1996) has recently reported that one of the antiangiogenic agents, thalidomide, promotes lymph node metastasis of rat prostate adenocarcinoma. Our present results have many points of similarity to Pollard's results. He speculates that the increased incidence of metastases due to thalidomide might be in part attributed to an immunosuppressive action of thalidomide. AGM-1470 might have a similar influence on the immune system of the LY80 tumour-bearing Donryu rats. Thus, further studies are needed to clarify why the incidence and growth of metastasized regional lymph node in rats implanted with LY80 are increased by AGM-1470 and why the tumour vessel formation in lymph nodes is not suppressed by AGM-1470.

In conclusion, the results in the present study showed that AGM-1470 promotes lymph node metastasis of LY80 cells. This fact suggests that there is the possibility that, in some cancer patients, lymph node metastases might be enhanced by AGM-1470 in the same way. Accordingly, application of this agent alone to patients should be carried out with great caution.

### ACKNOWLEDGEMENTS

This study was supported by the Haruo Sato Fund for Yoshida Sarcoma and Ascites Hepatoma Memorial. The authors thank Ms H Oikawa for her technical assistance.

### REFERENCES

Antoine N, Daukandt M, Heinen E, Simar LJ and Castronovo V (1996) *In vitro* and *in vivo* stimulation of the murine immune system by AGM-1470, a potent angiogenesis inhibitor. *Am J Pathol* **148**: 393–398

Futami H, Iseki H and Yamaguchi K (1994) Inhibition of lymphatic metastasis of rat fibrosarcoma by an angiogenesis inhibitor, AGM-1470. *Proc Am Assoc Cancer Res* **35**: 184

Ingber D, Fujita T, Kishimoto S, Sudo K, Kanamaru T, Brem H and Folkman J (1990) Synthetic analogues of fumagillin that inhibit angiogenesis and suppress tumour growth. *Nature* **348**: 555–557

Kurebayashi J, Kurosumi M, Dickson RB and Sonoo H (1994) Angiogenesis inhibitor o-(chloroacetyl-carbamoyl) fumagillol (TNP-470) inhibits tumor angiogenesis, growth and spontaneous metastasis of MKL-4 human breast cancer cells in female athymic nude mice. *Breast Cancer* **1**: 109–115

Kusaka M, Sudo K, Fujita T, Marui S, Itoh F, Ingber D and Folkman J (1991) Potent antiangiogenic action of AGM-1470: comparison to the fumagillin parent. *Biochem Biophys Res Commun* **174**: 1070–1076

McLeskey SW, Zhang L, Trock BJ, Kharbanda S, Liu Y, Gottardis MM, Lippman ME and Kern FG (1996) Effects of AGM-1470 and pentosan polysulphate on tumorigenicity and metastasis of FGF-transfected MCF-7 cells. *Br J Cancer* **73**: 1053–1062

Pollard M (1996) Thalidomide promotes metastasis of prostate adenocarcinoma cells (PA-III) in L-W rats. *Cancer Lett* **101**: 21–24

Tanaka T, Konno H, Matsuda I, Nakamura S and Baba S (1995) Prevention of hepatic metastasis of human colon cancer by angiogenesis inhibitor TNP-470. *Cancer Res* **55**: 836–839

Toi M, Takayanagi T, Souma R and Tominaga T (1994) Inhibition of vascular endothelial growth factor induced cell growth by an angiogenesis inhibitor AGM-1470 in capillary endothelial cells. *Oncol Rep* **1**: 423–426

Yamaoka M, Yamamoto T, Masaki T, Ikeyama S, Sudo K and Fujita T (1993*a*) Inhibition of tumor growth and metastasis of rodent tumors by the angiogenesis inhibitor o-(chloroacetyl-carbamoyl) fumagillol (TNP-470; AGM-1470). *Cancer Res* **53**: 4262–4267

Yamaoka M, Yamamoto T, Ikeyama S, Sudo K and Fujita T (1993*b*) Angiogenesis inhibitor TNP-470 (AGM-1470) potently inhibits the tumor growth of hormone-independent human breast and prostate carcinoma cell lines. *Cancer Res* **53**: 5233–5236

Yanase T, Tamura M, Fujita K, Kodama S and Tanaka K (1993) Inhibitory effect of angiogenesis inhibitor TNP-470 on tumor growth and metastasis of human cell lines *in vitro* and *in vivo*. *Cancer Res* **53**: 2566–2570

© Cancer Research Campaign 1997